# Lean management of crisis response and post-pandemic outcomes in US primary care practices

**DOI:** 10.1186/s12913-026-14968-3

**Published:** 2026-06-19

**Authors:** Dorothy Y. Hung, Stephen M. Shortell, Elicica Morris, Catalina Y. Quach, Elina Reponen, Thomas Rotter, Agustin Perez-Araos, Karen E. Schifferdecker

**Affiliations:** 1 Department of Health Policy and Management, Center for Lean Engagement and Research, School of Public Health, University of California, Berkeley, CA USA; 2 HUS Shared Group Services, Customer Relations, Helsinki University Hospital, Helsinki, Finland; 3 Health Quality Program, School of Nursing, Queens University, Kingston, ON Canada; 4 Department of Industrial Engineering, School of Engineering, Monterrey Tech, Guadalajara, Mexico; 5 Department of Community & Family Medicine, The Dartmouth Institute, Geisel School of Medicine, Dartmouth College, Hanover, NH USA

**Keywords:** Lean management, Quality/Process improvement, Primary care practices, National survey data, Covid-19 response, Incident command system, Clinician burnout, Financial performance, Workforce shortages, Multivariate logistic regression

## Abstract

**Background:**

Primary care forms the basis for valued outcomes in health systems, including population health, high quality care, and reduced health expenditures. However, the primary care system in the United States (US) faces many difficulties, including financial pressures and clinician burnout that accelerated following the Covid-19 pandemic. This study examines the ability of primary care practices to adapt to post-pandemic challenges using a lean management system for quality and process improvement.

**Methods:**

We used data from the National Survey of Healthcare Organizations and Systems II, a nationally representative sample of 1,245 physician practices located across the US. We first described the status of lean implementation among these primary care practices. We then conducted bivariate analyses followed by multivariate logistic regressions to examine relationships between lean implementation and outcomes of interest, including pandemic response activities, financial performance after the pandemic, and impacts of workforce shortages on patient care.

**Results:**

Practices that adopted lean management were significantly larger in size (*p* < 0.05), affiliated with a health system or federally qualified health center (*p* < 0.0001), had a higher percentage of revenue from Medicaid (*p* < 0.0001) but lower percentage from Medicare insurance (*p* < 0.05), participated in more capitated payment arrangements and Accountable Care Organization contract types (*p* < 0.01), and were more evenly distributed across geographic regions of the country (*p* < 0.001). Controlling for these contextual features, we found that lean practices were more likely to participate in an incident command system for pandemic response (OR = 2.45, 95% CI: 1.24–4.82), engage in efforts to address clinician burnout (OR = 1.29, 95% CI: 1.03–1.60), and report stronger financial performance after the pandemic (OR = 2.57, 95% CI: 1.40–4.73). Additionally, after adjusting for all other lean processes, practice use of rapid improvement events (OR = 0.20, 95% CI: 0.06–0.70) and real-time data analysis tools (OR = 0.31, 95% CI: 0.11–0.90) was associated with lower reported impacts of workforce shortages on patient care.

**Conclusion:**

Health systems continue to face challenges, making them vulnerable to major disruptions as experienced with the recent pandemic. Lean management may enhance organizational resilience and theability of primary care practices to respond to rapidly evolving environments.

**Supplementary Information:**

The online version contains supplementary material available at 10.1186/s12913-026-14968-3.

## Introduction

Primary care forms the basis for valued outcomes in health systems, including population health, higher quality care, and lower health expenditures [1, 2]. Yet financial and clinical pressures in the United States (US) have overstretched a primary care system that receives 5–7% of total healthcare spending compared with 14% in other developed nations, and where primary care providers constitute approximately one-third of the physician workforce, far below the recommended 40% needed for optimal outcomes [3]. These challenges increased with the recent pandemic that accelerated existing financial strain, employee burnout, and workforce shortages across care delivery systems. At the same time, studies conducted during the pandemic found that organizations with a lean management system showed high levels of resilience and agility in responding to such challenges [4–7]. 

Lean management is a sociotechnical approach to quality and process improvement that empowers staff with skills, tools, and resources needed to identify root causes of problems and implement needed changes [8, 9]. In primary care, lean process redesign led to increased efficiency among clinical teams, more timely care delivery, higher patient satisfaction, and enhanced work experiences among physicians and staff [10–14]. In the hospital setting, lean implementation has been linked to improved financial indicators and increased patient safety and quality measures, including reduced errors and lower 30-day readmission rates [15–17]. Collectively, this research is suggestive of the potential for lean management to enhance organizational performance while fostering supportive workflows to address challenges in rapidly evolving environments.

This study uses national survey data to examine associations between lean implementation, public health emergency (PHE) response, and post-pandemic outcomes in primary care practices located across the US. Specifically, we examine relationships between practice use of lean management and pandemic response activities such as participating in an incident command system, engaging in efforts to address clinician burnout, and restructuring staffing needs. We also investigate relationships between lean implementation, financial performance ratings after the pandemic, and perceived impacts of workforce shortages on patient care. Importantly, this study contributes to our understanding of primary care practices’ ability to adapt to major challenges using a lean management system for continuous improvement in care delivery settings.

## Methods

### Data source

Our study leverages cross-sectional data from the National Survey of Healthcare Organizations and Systems (NSHOS II), a nationally representative sample of 1,245 physician practices drawn from the IQVIA OneKey database [18, 19]. Further details of the NSHOS sampling and weighting methods are provided in an appendix. The NSHOS contains practices stratified by ownership (e.g., independent practice, integrated health system) with primary care physicians and advanced practice clinicians specializing in family medicine, internal medicine or geriatrics [20]. Practice leaders responded to a range of survey questions between June 2022-February 2023 on topics such as: practice characteristics and infrastructure, insurance mix and involvement in alternative payment arrangements, existing systems for clinical or operational quality improvement, and practice-level responses to the Covid-19 pandemic.

### Measures

#### Key independent variables

As described in Table 1, main independent variables were derived from practice use of any of six lean management processes: (1) A3 thinking/problem solving; (2) Value stream mapping (VSM); (3) Huddles; (4) Standard work/Daily management system; (5) Kaizen rapid improvement events; and (6) Analysis tools for quality improvement (e.g., Pareto charts). We first constructed a binary variable indicating practice use of any of the six lean management processes. We also created a summary variable indicating the extent of implementation measured as the number of lean processes used by a practice, ranging from 0 to all 6. Finally, we examined the six individual processes separately, each binary coded as 1 (“Yes”) or 0 (“No”) indicating practice use of each specific lean management technique.

**Table 1:** Description of lean management processes

*Lean Process*	*Description*
1. A3 Thinking / Problem Solving	Structured approach to problem solving in which a strategy to improve a problem is summarized concisely in a single document, historically a single sheet of A3 paper sized 11 × 17 inches. A3 reports typically include a problem definition, description of the current condition, a goal or target condition, root cause analysis, interventions or recommendations, and an implementation and sustainability plan. [37]
2. Value Stream Mapping	Technique that documents each step of a work process (e.g., patient rooming, delivery of a service or procedure) to understand the current state of operations, identify unnecessary or wasteful points in the process, and design a more ideal future state or workflow. [38]
3. Huddles	Short, focused meetings among team members to discuss key issues, coordinate care, and ensure alignment on patient needs, safety protocols, and workflow priorities. Meetings occur daily or weekly and are typically brief, lasting 10–15 min. A tiered huddle structure refers to huddles occurring at all levels of the organization to facilitate information sharing and dissemination. [39]
4. Standard Work / Daily Management System	Standardized work practices enabling staff and leaders to accomplish routine activities on a regular cadence in alignment with organizational goals. [4]
5. Kaizen Rapid Process Improvement Events	Rapid iterative tests of change, also called rapid process improvement workshops (RPIWs), to solve complex problems. Events typically last a few days with the goal of designing workflow changes and rapid cycle experiments to test viable solutions to problems. [40, 41]
6. Analysis Tools for Quality Improvement	Real-time data analysis tools to regularly monitor performance on operational or clinical targets. Examples include pareto charts that visually display data to focus improvement efforts on the most influential factors hindering performance, and statistical process control to distinguish between common vs. special cause variations indicating that a process is statistically aberrant. [42, 43]

#### Dependent variables

Study outcomes of interest included: practice engagement in various PHE response activities during the Covid-19 pandemic, financial performance ratings following the pandemic, and reported impact of workforce shortages on patient care. The NSHOS II assessed three types of response activities commonly observed during the pandemic via the following question, “In your practice’s response to Covid-19, did your practice do any of the following: 1. Participate in an incident command system; 2. Implement a process to address clinician burnout; 3. Layoff staff or reduce staff work hours?” Response options provided on the survey were binary coded as 1 (“Yes”) or 0 (“No”).

The survey also assessed ratings of financial performance at the time of survey and perceived impact of workforce shortages on patient care. The first item was assessed as follows, “Please rate how your practice is doing financially now: Poor, Fair, Good, or Very Good.” This was constructed as a binary variable with a response of “Very Good” coded as 1, and all other performance ratings coded as 0. The second item was assessed by the following question, “How much are clinician and staff shortages within your practice impacting patient care: Not at All, A Little, Some, or Quite a Lot?” This was queried separately for clinicians and staff with “Not at All” coded as 1 and all other responses coded as 0. Both items were subjective in nature as they were based on the accounts provided by survey respondents.

#### Covariates

We examined each practice’s organizational and regional characteristics with practice size and ownership sourced from the IQVIA OneKey database [18]. Practice size was represented by a categorical variable assessing the number of physicians working within the practice, defined as a single location practice: solo (1 physician), small (2–9 physicians), medium (10–20 physicians), or large (21 + physicians). Practice ownership consisted of the following distinct categories: independently owned, large physician group, hospital, healthcare system, or federally qualified health center (FQHC). Independent practice association (IPA) affiliation and acceptance of capitated payment were both assessed using binary response options. Insurance mix comprised of annual patient care revenue from various sources: commercial insurance; Medicare for older adults, including dual beneficiaries; Medicaid for low-income patients; and uninsured/self-pay/other (e.g., Tricare, VA, worker’s compensation). Response options included five categories analyzed on a scale reflecting increasing revenue from each insurance type.

We assessed participation in accountable care organizations (ACO) as the number of different payer types for which a practice held shared-risk ACO contracts, including contractual arrangements with Medicare, Medicaid, and commercial payers. The practice’s rural-urban continuum code (RUCA) was based on a practice’s business address and included classifications of: metropolitan, micropolitan, or rural/small town. Similarly, geographic regions were assigned based on the practice’s business address and categorized according to the Eastern, Southern, Midwest, or Western regions of the US.

### Statistical analysis

We first used t-tests and chi-square tests to describe differences in the organizational and geographic features of practices that implemented lean versus those that did not. We also examined raw unadjusted associations between each of the study outcomes and lean implementation assessed in three ways: (1) adoption of any lean management process vs. none at all; (2) extent of adoption as measured by the number of lean processes in place; and (3) adoption of each specific type of lean process. Based on statistically significant findings, we proceeded with multivariate logistic regressions on select outcomes to further examine their associations with lean implementation, controlling for practice size, ownership, annual insurance revenue, payment arrangements, RUCA, and geographic region. A last set of regressions examined the use of each specific type of lean management process, controlling for all other processes that may be used in a practice (i.e., all were included in the were included in the same model to adjust for potentially confounding effects). Finally, a Hochberg step-up correction was applied to model results, adjusting all p-values upward to account for potential multiplicity of outcomes. All statistical analyses incorporated both sampling and nonresponse weights and were conducted using R studio software (Integrated Development Environment for R, Boston, MA).

## Results

Following Table 1 description of lean management processes, Table 2 describes the status of lean implementation in this national sample of primary care practices. Differences between practices that adopted any lean management processes versus none are displayed in terms of their organizational and geographic features. Specifically, lean adopters tended to be larger in size (*p* < 0.05), affiliated with a health system or federally qualified health center (*p* < 0.0001), and have a higher percentage of patient revenue from Medicaid (*p* < 0.0001), but lower percentage of revenue from Medicare (*p* < 0.05). Lean practices were also more likely to report having capitated payment (*p* < 0.01), participate in a wider range of ACO contract types (*p* < 0.0001), and were more evenly distributed across geographic regions of the US (*p* < 0.001).

**Table 2:** Lean implementation status in US primary care practices (*N* = 1,245)

	Any Lean Management Processes (*n* = 886)	No Lean Management Processes (*n* = 310)	*p*-value
**Number of Lean Processes Implemented** Range: 0–6, Mean (SD)	2.03 (1.28)	-	-
**Type of Lean Management Process**			
A3 Thinking / Problem Solving	104 (11.7%)	-	-
Value Stream Mapping	105 (11.9%)	-	-
Huddles	805 (90.9%)	-	-
Standard Work / Daily Management System	453 (51.1%)	-	-
Kaizen Rapid Process Improvement Events	103 (11.6%)	-	-
Analysis Tools for Quality Improvement (e.g., Pareto charts, Statistical process control)	233 (26.3%)	-	-
**Practice Size**			0.031
Solo	40 (4.6%)	23 (7.4%)	
Small (2–9 physicians)	618 (70.9%)	229 (74.1%)	
Medium (10–19 physicians)	114 (13.1%)	25 (8.1%)	
Large (20 + physicians)	100 (11.5%)	32 (10.4%)	
**Ownership**			< 0.0001
Independently Owned	214 (24.5%)	133 (43.8%)	
Physician Group	77 (8.8%)	31 (10.2%)	
Hospital	76 (8.7%)	26 (8.6%)	
Health System	298 (34.1%)	72 (23.7%)	
FQHC	207 (23.7%)	39 (12.8%)	
Other	1 (0.1%)	3 (1.0%)	
**IPA Affiliation** (% Yes)	172 (19.7%)	76 (24.9%)	0.054
**Insurance: Annual Patient Care Revenue** ^**a**^ Mean (SD)			
Commercial	3.12 (1.00)	3.18 (0.91)	0.373
Medicare (including duals)	3.01 (0.84)	3.15 (0.85)	0.022
Medicaid	2.25 (1.16)	1.88 (1.08)	< 0.0001
Uninsured/Self-pay/Other (Tricare, VA, Worker’s Compensation, etc.)	1.44 (0.78)	1.37 (0.75)	0.215
**Capitation** (% Yes)	390 (47.7%)	104 (36.6%)	0.001
**ACO Participation** ^**b**^ Mean (SD)	1.80 (1.2)	1.46 (1.2)	< 0.0001
**Rural-Urban Continuum Code** ^**c**^			0.716
Metropolitan	714 (82.8%)	245 (80.9%)	
Micropolitan	89 (10.3%)	36 (11.9%)	
Rural/Small Town	59 (6.8%)	22 (7.3%)	
**Geographic Region**			0.001
East	182 (20.5%)	48 (15.5%)	
Midwest	242 (27.3%)	77 (24.8%)	
South	233 (26.3%)	120 (38.7%)	
West	229 (25.9%)	65 (21.0%)	

Note: Weighted percentages (unweighted counts) are reported to adjust for missing data

FQHC: Federally Qualified Health Center; IPA: Independent Practice Association; ACO: Accountable Care Organization

^a^Annual patient care revenue ranging from 1 (less than 10%) to 5 (greater than 80%)

^b^Number of ACO payer types

^c^Classification for practice’s business address

Tables 3 and 4 show raw bivariate relationships between all study outcomes and lean implementation, prior to controlling for organizational and geographic characteristics. Relationships between study outcomes and each of the six lean management processes are also shown, prior to controlling for all other lean processes that may exist in a practice. In these unadjusted analyses, there were strong bivariate associations between lean management and crisis response activities such as participating in incident command and addressing clinician burnout (*p* < 0.0001). There were also statistically significant associations with financial performance ratings (*p* < 0.05) and reported impact of clinician shortages on patient care (*p* < 0.01). Among practices that implemented lean, we found varied statistical significance in the bivariate relationships between individual lean processes and post-pandemic outcomes.

**Table 3:** Unadjusted associations between lean implementation and pandemic response activities (*N* = 1,245)

	Incident Command System			Clinician Burnout			Staff Layoffs or Reduced Hours		
	Participation *(n = 512)*	No Participation *(n = 699)*	*p*-value	*Effort to Address (n = 389)*	*No Effort to Address (n = 828)*	*p*-value	Occurrence *(n = 467)*	No Occurrence *(n = 752)*	*p*-value
**Lean Implementation**									
Any Lean Management Processes (% Yes)	430 (85.8%)	447 (65.5%)	< 0.0001	327 (85.6%)	555 (68.7%)	< 0.0001	333 (73.0%)	550 (74.8%)	0.490
Number of Lean Processes [Range: 0–6, Mean (SD)]	2.06 (1.59)	1.20 (1.11)	< 0.0001	2.20 (1.58)	1.23 (1.25)	< 0.0001	1.50 (1.43)	1.51 (1.42)	0.846
**Type of Lean Management Process**									
A3 Thinking / Problem Solving	72 (14.3%)	30 (4.3%)	< 0.0001	55 (14.4%)	48 (5.9%)	< 0.0001	41 (8.9%)	62 (8.4%)	0.760
Value Stream Mapping	79 (15.6%)	26 (3.8%)	< 0.0001	63 (16.4%)	44 (5.4%)	< 0.0001	48 (10.4%)	59 (7.9%)	0.146
Huddles	415 (81.1%)	402 (57.9%)	< 0.0001	314 (80.7%)	508 (61.7%)	< 0.0001	305 (65.6%)	518 (69.2%)	0.196
Standard Work / Daily Management System	253 (49.7%)	202 (29.2%)	< 0.0001	210 (54.0%)	248 (30.2%)	< 0.0001	177 (38.2%)	281 (37.7%)	0.867
Kaizen Rapid Improvement Events	76 (15.1%)	27 (3.9%)	< 0.0001	56 (14.6%)	48 (5.9%)	< 0.0001	34 (7.4%)	70 (9.5%)	0.212
Analysis Tools for Quality Improvement (e.g., Pareto charts, Stat. process control)	156 (30.8%)	78 (11.3%)	< 0.0001	119 (30.8%)	116 (14.2%)	< 0.0001	88 (19.1%)	147 (19.7%)	0.798

Note: Table reports weighted percentages (unweighted counts)

**Table 4:** Unadjusted associations between lean implementation and post-pandemic outcomes

	Financial Performance			Clinician Shortage			Non-Clinician Shortage		
	Very Good *(n = 193)*	All Other *(n = 1012)*	*p*-value	Impacted Care *(n = 933)*	No Impact *(n = 280)*	*p*-value	Impacted Care *(n = 1072)*	No Impact *(n = 139)*	*p*-value
**Lean Implementation**									
Any Lean Management Processes (% Yes)	146 (78.5%)	720 (73.0%)	0.115	692 (76.4%)	182 (66.4%)	0.001	777 (74.5%)	94 (69.9%)	0.225
Number of Lean Processes [Range: 0–6, Mean (SD)]	1.74 (1.55)	1.44 (1.37)	0.017	1.56 (1.43)	1.30 (1.29)	0.005	1.50 (1.40)	1.42 (1.42)	0.538
**Type of Lean Management Process**									
A3 Thinking / Problem Solving	22 (11.7%)	74 (7.4%)	0.049	83 (9.1%)	15 (5.4%)	0.052	89 (8.5%)	9 (6.5%)	0.434
Value Stream Mapping	21 (11.1%)	79 (7.9%)	0.149	85 (9.3%)	17 (6.1%)	0.101	91 (8.6%)	11 (8.0%)	0.791
Huddles	137 (71.7%)	668 (66.5%)	0.161	654 (70.7%)	159 (57.2%)	< 0.0001	729 (68.6%)	81 (58.7%)	0.020
Standard Work / Daily Management System	84 (44.0%)	364 (36.4%)	0.048	350 (37.9%)	103 (37.3%)	0.857	399 (37.6%)	53 (39.0%)	0.757
Kaizen Rapid Improvement Events	16 (8.5%)	84 (8.4%)	0.975	83 (9.1%)	17 (6.1%)	0.121	90 (8.6%)	9 (6.5%)	0.413
Analysis Tools for Quality Improvement (e.g., Pareto charts, Stat. process control)	48 (25.5%)	183 (18.3%)	0.022	185 (20.1%)	48 (17.5%)	0.326	202 (19.1%)	31 (22.8%)	0.310

Note: Table reports weighted percentages (unweighted counts)

Tables 5 and 6 present selected multivariate logistic regression results, controlling for organizational and geographic characteristics. Practices that implemented lean, models also control for all other lean processes present in a practice. Table 5 supports previous bivariate results by showing that lean management was associated with more than twice the odds of participating in incident command for crisis response during the pandemic (OR = 2.45, 95% CI: 1.24–4.82). Moreover, the extent of lean implementation as reflected by the number of processes in place was positively related to participation in incident command (OR = 1.65, 95% CI: 1.32–2.06). Among these processes, the most significant relationship was found with practice use of real-time data analysis for quality improvement (OR = 3.25, 95% CI: 1.56–6.74). Similarly, results suggest that practices with a high degree of lean implementation were more apt to engage in efforts to address clinician burnout during the pandemic (OR = 1.29, 95% CI: 1.03–1.60). In particular, analysis tools such as pareto charts and statistical process control were associated with over twice the odds of addressing burnout (OR = 2.37, 95% CI: 1.14–4.93).

**Table 5:** Logistic regression results: pandemic response activities^a^

	Participation in Incident Command System		Efforts to Address Clinician Burnout	
	Odds Ratio	95% CI	Odds Ratio	95% CI
**Lean Implementation**				
Any Lean Management Processes (% Yes)	2.45**	**[1.24**,** 4.82]**	1.06	[0.51, 2.18]
Number of Lean Processes ^b^ (Range: 0–6)	1.65***	**[1.32**,** 2.06]**	1.29*	**[1.03**,** 1.60]**
**Type of Lean Management Process**				
A3 Thinking / Problem Solving	0.37	[0.12, 1.16]	0.73	[0.22, 2.49]
Value Stream Mapping	2.05	[0.92, 4.61]	1.18	[0.46, 2.97]
Huddles	1.56	[0.79, 3.09]	0.79	[0.39, 1.59]
Standard Work / Daily Management System	1.44	[0.74, 2.77]	1.26	[0.68, 2.36]
Kaizen Rapid Improvement Events	4.76*	**[1.31. 17.24]**	2.49	[0.82, 7.56]
Analysis Tools for Quality Improvement (e.g., Pareto charts, Stat. process control)	3.25**	**[1.56**,** 6.74]**	**2.37**	**[1.14**,** 4.93]**

Note: For each pandemic response activity, the first two table rows present results from two separate regressions with “Any Lean Management Processes” (Yes/No) and “Number of Lean Processes” as the main independent variable in each model, respectively. All other table content presents results of a single regression model that estimates associations between each type of lean process and response activity, controlling for all other lean processes

All regressions adjust for: practice size, ownership, IPA affiliation, insurance (annual patient care revenue), capitation, ACO participation, rural-urban continuum, and geographic region

^a^ Occurrence of staff layoffs/reduced hours was not associated with lean implementation or use of specific lean processes (data not shown). Null results from these multivariate logistic regressions are available upon request

^b^ Regression results show associations between practice use of each additional lean process and engagement in pandemic response activities

Hochberg adjusted p-values: *p < 0.10, **p < 0.05, ***p < 0.01

**Table 6:** Logistic regression results: post-pandemic financial performance and impact of workforce shortages

	Financial Performance		Impact of Clinician Shortages on Care		Impact of Non-Clinician Shortages on Care^a^	
	Odds Ratio	95% CI	Odds Ratio	95% CI	Odds Ratio	95% CI
**Lean Implementation**						
Any Lean Management Processes (% Yes)	2.57**	**[1.40**,** 4.73]**	1.67	[0.93, 3.02]	-	-
Number of Lean Processes ^b^ (Range: 0–6 processes)	1.36**	**[1.08**,** 1.71]**	1.00	[0.80, 1.25]	-	-
**Type of Lean Management Process**						
A3 Thinking / Problem Solving	0.90	[0.25, 3.32]	1.73	[0.47, 6.40]	3.21	[0.53, 19.5]
Value Stream Mapping	0.49	[0.17, 1.42]	2.24	[0.73, 6.91]	3.83	[0.63, 23.6]
Huddles	3.28***	**[1.72**,** 6.25]**	1.52	[0.83, 2.80]	0.82	[0.43, 1.60]
Standard Work / Daily Management System	0.99	[0.42, 2.32]	1.29	[0.65, 2.57]	0.80	[0.36, 1.77]
Kaizen Rapid Improvement Events	1.55	[0.35, 6.90]	0.20*	**[0.06**,** 0.70]**	0.55	[0.09, 3.29]
Analysis Tools for Quality Improvement (e.g., Pareto charts, Stat. process control)	2.05	[0.88, 4.80]	0.55	[0.23, 1.28]	**0.31**	**[0.11**,** 0.90]**

Note: For each post-pandemic outcome, the first two table rows present results from two separate regressions with “Any Lean Management Processes” (Yes/No) and “Number of Lean Processes” as the main independent variable in each model, respectively. All other table content presents results of a single regression model that estimates associations between each type of lean process and outcome, controlling for all other lean processes

All regressions adjust for: practice size, ownership, IPA affiliation, insurance (annual patient care revenue), capitation, ACO participation, rural-urban continuum, and geographic region

^a^ Impact of non-clinician shortages on patient care was not associated with lean implementation (data not shown). Null results from these multivariate logistic regressions are available upon request

^b^ Regression results show associations between practice use of each additional lean process and reporting of post-pandemic outcomes

Hochberg adjusted p-values: *p < 0.10, **p < 0.05, ***p < 0.01

According to Table 6, primary care practices that adopted lean management had more than double the odds of reporting very good financial performance at the time of survey (OR = 2.57, 95% CI: 1.40–4.73). Specifically, use of team huddles was associated with higher performance ratings after adjusting for the use of all lean processes (OR = 3.28, 95% CI: 1.72–6.25). When examining perceived impact of workforce shortages on patient care, there was no significant difference between practices that reported adopting lean management versus those that did not. Among those that did, the impact of clinician shortages on patient care was found to be slightly lower in practices that engaged in kaizen improvement (OR = 0.20, 95% CI: 0.06–0.70). Similarly, in practices that implemented lean, use of real-time analysis tools was slightly associated with lower perceived impact of non-clinician shortages on patient care (OR = 0.31, 95% CI: 0.11–0.90).

## Discussion

This cross-sectional study highlights several potential roles of a lean management system in supporting crisis response. First, familiarity with lean processes may facilitate response mechanisms that require immediate feedback and action. For example, the systematic approach to conducting kaizen rapid improvement, which utilizes Plan Do Check Act (PDCA) cycles to iteratively develop solutions to problems, is synergistic with the steps of incident command outlined by Phibbs and Snawder: [21] (1) Create a set of objectives and goals (Plan); (2) Develop tactics and viable solutions while ensuring appropriate resources (Do); (3) Review the plan to confirm it is sound (Check); (4) Execute the plan, receive feedback and communicate updates, and ensure accountability (Act). Our study aligns with this approach by finding greater participation in incident command among practices that engaged in kaizen rapid cycle improvement methodology. These efforts often rely on timely measurement and monitoring of performance using real-time analysis tools for quality improvement, which were also associated with practice engagement in incident command systems. While our cross-sectional data cannot establish causality, these findings suggest that practice agility and the capability to implement needed changes, aided by lean processes to support such adjustments, can facilitate quick response to evolving environments.

In our study, practices that adopted lean management were also more likely to address clinician burnout during the pandemic. This finding reflects a lean guiding principle of respecting every individual—not only patients but also the providers who care for them [22]. Another lean guiding principle, leading with humility, is commonly recognized as a key leadership attribute in lean organizations. Adherence to both of these guiding principles creates a culture that is sensitive to workforce wellbeing, and aligns with reports that feeling valued by leaders and believing concerns will be acted upon are protective against burnout [23]. In addition to supporting wellness among individuals, prior research highlights the need to facilitate progress at the broader system level [24]. According to our current study results, efforts to address burnout were higher in practices that used analysis tools such as pareto charts and statistical process control for quality improvement purposes. This indicates a proactive approach to identifying and addressing systemic issues, including those that may impact professional wellbeing.

We examined perceptions of financial performance after the pandemic and found more favorable ratings among practices that adopted lean management. While this performance measure is subjective as actual performance cannot be verified with our data, we note prior research findings that a lean approach to continuous improvement helped organizations achieve a range of benefits such as improved resource utilization, elimination of wasteful processes, enhanced productivity, and minimization of errors [25], all of which can have positive impacts on financial status. Lean management has also been shown to directly improve cost and revenue metrics; [15–17, 26–27] in particular, work standardization and reduced variability due to process improvements were identified as key to reducing costs [28]. Hence while our assessment of financial performance was based on perceptions, the direction of association found between lean and practice finances is consistent with prior work in this area.

Additionally, based on our descriptive analysis, practices that implemented lean such as FQHCs and those with high Medicaid patient loads were more likely to have capitated payment or participate in various ACO contracts. These incentive structures are also designed to eliminate waste, boost efficiency and improve value. Such safety nets also often participate in organized collaboratives and centralized quality initiatives to ensure high value care is provided to socioeconomically disadvantaged patients. Our finding reinforces a prior study of safety net clinics, including FQHCs, that expanded their practice infrastructure and capacity to care for vulnerable populations after the pandemic [29]. That study along with our current findings may be partially driven by safety net payment arrangements that reward population-based management along with high-value care delivery.

Among practices that implemented lean management techniques, and when adjusting for all other lean processes, we found that use of huddles among primary care teams was associated with more positive ratings of financial performance. In general, huddles are designed to promote effective communication by fostering collaboration and aligning members toward common goals. In previous research conducted during the pandemic [4], health system leaders commonly cited their tiered huddle structure as critical for rapid communication and dissemination of new information across the enterprise. This had significant bearing on daily operations with direct implications for system finances. In our current sample of physician practices, daily huddles may have also facilitated information transfer and instruction to care teams during the pandemic. This level of teamwork likely led to positive perceived outcomes, though we are unable to assess whether these perceptions reflected reality.

Finally, we note the null finding on lean implementation and workforce layoffs/reduced hours among primary care staff. This finding occurs in the context of a broader Covid-19 literature that describes intensified workforce challenges [30] in the form of increased layoffs and reduction in work hours [31–34]. Our lack of statistically significant findings may reflect a no-layoff philosophy of lean implementation that aims to create an environment of psychological safety, and where staff feel comfortable engaging in improvement activities without fear of improving themselves out of employment [35, 36]. Since our research did not involve other data sources to investigate the nuances of staffing changes, additional studies can be undertaken to further explore this finding. As for any subsequent impact of workforce shortages on patient care, the adjusted regression results suggest that engaging in kaizen initiatives and data analysis for quality improvement may have protective effects, controlling for the use of all other lean processes.

Some limitations of this study include the inability of our cross-sectional survey data to establish causality regarding lean’s contribution to improved performance. It may be that practices with stronger leadership, greater resources, or more stable baseline performance were more likely to adopt lean methodology while also reporting more favorable outcomes. Second, there may be potential for selective satisficing, i.e., falsely reporting use of lean as a fashionable management practice, when in reality, lean tools are rarely or not at all used. Yet as lean survey items constituted one small piece of the full NSHOS that included many other topics, ranging from patient-reported measures and motivational interviewing to shared decision making and care of complex high-needs patients, there may be less reason to suspect false reporting or selective satisficing. Moreover, based on our descriptive analysis, practices that reported implementing lean reported having an average 2.03 lean processes in place. One might expect the number of processes to be on the higher end of the spectrum, perhaps closer to the full count of 6 lean processes, if fashionable for leaders to claim their clinic had implemented lean. Third, similar to the previous point regarding a need to better understand the nuances of staffing changes, some of our findings on specific lean techniques such as kaizen, huddles, and real-time analysis tools would benefit from a clearer context for how these processes were used. Lean techniques may not have inherent effects themselves, but rather, how they are applied in specific organizational contexts may be a key factor associated with positive outcomes. Finally, regarding our study’s practical and financial implications, caution should be exercised as certain outcomes such as financial performance and impact of workforce shortages are based on self-reports rather than objective measures. As such, these findings reflect perceptions rather than demonstrated operational or financial effects.

## Conclusion

Health systems continue to face challenges, making them vulnerable to major disruptions as experienced with the recent Covid-19 pandemic. Our findings suggest that when implemented comprehensively, lean as a sociotechnical approach to quality and process improvement may enhance crisis response by creating a potentially more agile, resilient work environment. However, further research is needed to specify further research is needed to specify the mechanisms by which lean can be used to address challenges during an emergency. Additionally, qualitative research may help better understand the nature of changes and associated relationships found in our study. Future investigations might explore how lean management can enable physician practices to address important issues they face in their current environments.

## Supplementary Information

Below is the link to the electronic supplementary material.

Supplementary Material 1

## Data Availability

The National Survey of Healthcare Organizations and Systems (NSHOS II) data and materials can be made available upon request.
