# Supplementary material for: Lean management of crisis response and post-pandemic outcomes in US primary care practices
Source: BMC Health Serv Res. 2026 Jun 19;26:1181. doi: 10.1186/s12913-026-14968-3 (PMC13508409; doi:10.1186/s12913-026-14968-3)
Supplement: Supplementary file 1 [file 12913_2026_14968_MOESM1_ESM.docx]

**Appendix: National Survey of Healthcare Organizations & Systems for Physician Practices (NSHOS II) Sampling Description**

Similar to the NSHOS I previous study, we used stratified-cluster sampling of primary care practices, excluding pediatric practices. The sample consisted of prior study respondent practices (NSHOS I, see below) and a stratified random selection of additional practices based on federally qualified health center (FQHC) status, area deprivation, and ownership type (independent, medical group, system).

Our full sample frame came from IQVIA's commercially available OneKey database, which describes relationships among physicians, practices, hospitals, and healthcare systems. IQVIA uses the American Medical Association’s physician Masterfile, publicly available sources, and proprietary data collection strategies to develop the OneKey database. We identified primary and multispecialty care physician practices based on the 2022 population of practices in the US that had at least three primary care physicians. We defined physician practices as a single location practice. Primary care physicians included family medicine, geriatrics, internal medicine, or preventive medicine specialties.

We used a stratified-cluster sampling design to select physician practices. We stratified our sample based on ownership and composition structures including samples of both system-owned and independent physician practices and hospitals.

All analyses were performed using Stata, version 18.0 (StataCorp) and survey weights to account for the probability that a practice was sampled from the sampling frame of eligible practices (based on the 2022 population of practices in the US) and whether the practice responded to our survey.

Sampling and Survey Non-response Weights Description. Analyses were performed using survey weights to account for the probability that a practice was part of the original NSHOS I study sample or was sampled from the sampling frame of eligible practices (established based on the 2022 population of medical practices in the US), and whether the practice responded to our survey (to account for nonresponse). Because practices that were part of the prior study were retained with probability 1, their effective sampling probability was 1 with respect to the 2022 sampling frame for this NSHOS II study. For newly eligible practices, we included 100% of FQHCs and an almost equal number (166 or 167) of non-FQHCs (1000 total) for the six combinations of system-type and a binary variable indicating whether the Federal Information Processing Standards (FIPS) decile was 8. Thus, for our entire study sample, there were seven (six non-FQHCs and FQHCs) types of practices in total. For the newly eligible study sample, the sampling probabilities were simple proportions equal to the numerator divided by the number of practices in the original 2022 sampling frame (prior to knowing information regarding eligibility that was learned through the survey process) of the seven types of practices. Upon learning more about the practices after sampling them, some of the sampled practices were deemed to be ineligible. Of the sampled practices that were eligible, a certain fraction responded, yielding seven proportions of responders. Therefore, the joint probability of a practice being sampled, found to be eligible, and responding was estimated as the product of the corresponding three proportions for each of the seven types of practices. The inverses of these joint probabilities formed the marginal weights used in our chi-square test analyses.

NSHOS I respondent practices sampling: Selecting the sample. We used a Monte-Carlo algorithm to select the sample applying the following principles: (1) for every sampled system, multiple organizations (subsidiaries, hospitals, practices) will also be sampled; (2) the probability of sampling an organization (system or medical group) will increase with the number of hospitals or medical practices within it; (3) subsidiary integrated delivery systems are included only if their parent is sampled and if a complex system owns one or more subsidiary integrated systems, then at least one of these will be sampled; (4) if any system is sampled and has 4 or fewer practices or hospitals, all of these will be sampled; (5) if any system has 4 or more practices or hospitals, the probability of sampling will increase logarithmically from a minimum of 4 to a maximum of 10. The use of a Monte-Carlo algorithm was required to compute the sampling probability for each sampled organization, which enabled us to draw population-representative inferences.
